# Hepatic artery infusion chemotherapy using mFOLFOX versus transarterial chemoembolization for massive unresectable hepatocellular carcinoma: a prospective non-randomized study

**DOI:** 10.1186/s40880-017-0251-2

**Published:** 2017-10-23

**Authors:** Min-Ke He, Yong Le, Qi-Jiong Li, Zi-Shan Yu, Shao-Hua Li, Wei Wei, Rong-Ping Guo, Ming Shi

**Affiliations:** Department of Hepatobiliary Oncology, State Key Laboratory of Oncology in South China, Collaborative Innovation Center for Cancer Medicine, Sun Yat-sen University Cancer Center, 651 Dongfeng Road East, Guangzhou, 510060 Guangdong P. R. China

**Keywords:** Hepatocellular carcinoma, Hepatic artery infusion chemotherapy, Transarterial chemoembolization, mFOLFOX

## Abstract

**Background:**

Transarterial chemoembolization (TACE) is recommended as the standard care for unresectable hepatocellular carcinoma (HCC) at Barcelona Clinic Liver Cancer (BCLC) stage A–B. However, the efficacy of TACE on large (≥ 10 cm) stage A–B HCC is far from satisfactory, and it is proposed that hepatic artery infusion chemotherapy (HAIC) might be a better first-line treatment of this disease. Hence, we compared the safety and efficacy of HAIC with the modified FOLFOX (mFOLFOX) regimen and those of TACE in patients with massive unresectable HCC.

**Methods:**

A prospective, non-randomized, phase II study was conducted on patients with massive unresectable HCC. The protocol involved HAIC with the mFOLFOX regimen (oxaliplatin, 85 mg/m^2^ intra-arterial infusion; leucovorin, 400 mg/m^2^ intra-arterial infusion; and fluorouracil, 400 mg/m^2^ bolus infusion and 2400 mg/m^2^ continuous infusion) every 3 weeks and TACE with 50 mg of epirubicin, 50 mg of lobaplatin, 6 mg of mitomycin, and lipiodol and polyvinyl alcohol particles. The tumor responses, time-to-progression (TTP), and safety were assessed.

**Results:**

A total of 79 patients were recruited for this study: 38 in the HAIC group and 41 in the TACE group. The HAIC group exhibited higher partial response and disease control rates than did the TACE group (52.6% vs. 9.8%, *P* < 0.001; 83.8% vs. 52.5%, *P* = 0.004). The median TTPs for the HAIC and TACE groups were 5.87 and 3.6 months (hazard radio [HR] = 2.35, 95% confidence interval [CI] = 1.16–4.76, *P* = 0.015). More patients in the HAIC group than in the TACE group underwent resection (10 vs. 3, *P* = 0.033). The proportions of grade 3–4 adverse events (AE) and serious adverse events (SAE) were lower in the HAIC group than in the TACE group (grade 3–4 AEs: 13 vs. 27, *P* = 0.007; SAEs: 6 vs. 15, *P* = 0.044). More patients in the TACE group than in the HAIC group had the study treatment terminated early due to intolerable treatment-related adverse events or the withdrawal of consent (10 vs. 2, *P* = 0.026).

**Conclusions:**

HAIC with mFOLFOX yielded significantly better treatment responses and less serious toxicity than did TACE. HAIC might represent a feasible and promising first-line treatment for patients with massive unresectable HCC.

## Introduction

Hepatocellular carcinoma (HCC) is the third common cancer worldwide and the second leading cause of cancer death in China [1]. Currently, most guidelines recommend transarterial chemoembolization (TACE), a technique combining intra-arterial chemotherapy and selected embolization, as the standard of care for unresectable HCC at Barcelona Clinic Liver Cancer (BCLC) stage A–B [2–4]. However, the efficacy of TACE on large (≥ 10 cm) stage A–B HCC is far from satisfactory [5–7]. The disease control rate (DCR) was less than 50%, and the median overall survival was only 6.5–9.1 months [8–10]. One reason is that complete embolization of all tumor-feeding arteries is especially difficult for massive HCCs because these HCCs usually have multiple intrahepatic and/or extrahepatic collateral arterial supplies and arteriovenous fistulas. Moreover, after embolization for large tumors, patients are at a high risk of serious embolization-related adverse events (AEs), such as serious post-embolization syndrome, liver/renal dysfunction, and ectopic embolism. TACE-related death rates of 6.5–20% have been reported [9, 10].

Some researchers have proposed that continuous hepatic artery infusion chemotherapy (HAIC) might be a better first-line treatment than TACE for patients with massive HCCs [11, 12]. Recent researches suggest that chemotherapy plays an important role in TACE, and adding embolization might be more detrimental than beneficial for these patients [13–16]. Compared with TACE, HAIC provides more stable and sustained local delivery of chemotherapy agents [17]. Several cisplatin (DDP)-based HAIC regimens have been reported to provide an encouraging therapeutic efficacy on HCC [18–21]. Nevertheless, the DDP dose was limited by renal, neurological, and gastrointestinal toxicities [22]. In contrast, oxaliplatin (OXA) has been reported to be a better DNA synthesis inhibitor than cisplatin and have better synergism with 5-fluorouracil (5-FU) with a different toxicity profile [23–25]. Furthermore, two recent large multicenter studies evaluating OXA-based regimens as first-line systemic chemotherapy for advanced HCC demonstrated manageable toxicities and promising tumor responses [26, 27].

Hence, it is important to investigate whether OXA-based HAIC is a better first-line treatment than TACE for patients with massive unresectable HCC. However, to date, only a few preliminary studies have evaluated the efficacy and safety of OXA-based HAIC regimens compared with conventional TACE. One phase I study showed that OXA-based HAIC was well tolerable in HCC patients [28]. Additionally, another retrospective single-cohort study reported by Li et al. [29] showed that the combination use of OXA-based HAIC and conventional TACE is a safe and promising treatment for patients with HCCs larger than 10 cm in diameter. Here, we present results from a prospective, non-randomized, controlled study that assessed the efficacy and safety of HAIC with OXA, 5-FU, and leucovorin (LV) in patients with massive unresectable HCC. The modified FOLFOX6 (mFOLFOX6) regimen was used because the safety of HAIC with the mFOLFOX6 regimen has been documented in several phase I/II trials in patients with hepatic metastases from colorectal cancer [30, 31]. Our primary objective was to compare the responses of massive unresectable HCC to HAIC with FOLFOX with those to TACE. The secondary objective was to assess the time-to-radiological progression (TTP) and toxicity.

## Patients and methods

### Patients and study design

This prospective, non-randomized study was approved by the institutional review board of Sun Yat-sen University Cancer Center and was performed in accordance with the Declaration of Helsinki of 1975 as revised in 1983. The study was registered at http://ClinicalTrials.gov (No. NCT03048123). Between October 1, 2015 and October 1, 2016, consecutive patients with unresectable HCC treated at our institution were enrolled. Patients had to provide signed informed consent before enrollment in the study.

HCC was diagnosed based on the criteria used by the European Association for the Study of the Liver (EASL). All patients met the following criteria: (a) the sum of diameters of all lesions longer than 10 cm with the maximum lesion longer than 7 cm; (b) age between 18 and 75 years; (c) the tumor was not amenable to surgical resection or any other curative treatment; (d) platelet count ≥ 75,000/μL, hemoglobin ≥ 8.5 g/dL, total bilirubin ≤ 30 mmol/L, and serum albumin ≥ 32 g/L; and (e) the absence of cirrhosis or a cirrhotic status of Child–Pugh class A only. Patients were excluded from the study if they met any of the following criteria: (a) a previous history of treatment for HCC; (b) vascular invasion or distant metastasis; (c) severe underlying cardiac or renal diseases; or (d) a second primary malignancy. Each patient was informed of the details of the TACE and HAIC procedures, especially concerning the uncertain benefits and complication risks associated with HAIC, as well as other possible treatment options, such as systemic chemotherapy. The treatment choice of either HAIC or TACE was made at the patients’ request after a full discussion with our multidisciplinary treatment team, which included radiologists, surgeons, hepatologists, and oncologists.

### Treatment

TACE was performed according to our previously reported protocol [16]. Chemolipiodolization was performed using 50 mg of epirubicin (pharmorubicin; Pfizer, Wuxi, Jiangsu, China), 50 mg of lobaplatin (Hainan Changan International Pharmaceutical Co. Ltd., Haikou, Hainan, China), and 6 mg of mitomycin C (Zhejiang Hisun Pharmaceutical Co. Ltd., Taizhou, Zhejiang, China) mixed with 10 mL of lipiodol (Lipiodol Ultra-Fluide; Guerbet Laboratories, Aulnay Sous Bois, Paris, France). If necessary, up to 20 mL of additional pure lipiodol was injected. The injection was stopped when stasis of blood flow in the target artery was observed. Subsequently, embolization was performed with the injection of polyvinyl alcohol particles that were 300–500 μm in diameter through the catheter to reach stasis in the tumor-feeding artery. Repeated TACE was performed at intervals of 6 weeks.

In the HAIC group, patients were treated using a 3-week cycle regimen. A catheter was advanced into the hepatic artery according to our previously reported protocol [16]. A microcatheter was selectively placed into the feeding arteries of the tumor. The gastroduodenal artery was occluded by a coil when necessary. Then, the microcatheter was connected to the artery infusion pump to administer the following treatment: OXA, 85 mg/m^2^ intra-arterial infusion on day 1; LV, 400 mg/m^2^ intra-arterial infusion on day 1; and 5-FU, 400 mg/m^2^ bolus infusion on day 1 and 2400 mg/m^2^ continuous infusion over 46 h. After HAIC was completed, the indwelling catheter and the sheath were removed, and manual compression was performed to achieve hemostasis.

HAIC and TACE were discontinued when disease progression (including vascular invasion or the development of extrahepatic spread) or intolerable AEs occurred or when the patient was eligible for another treatment (surgical resection) or withdrew consent. Additionally, the study treatment was suspended when the following conditions occurred: technical difficulty in repeating the treatment (stenosis or occlusion of the tumor-feeding artery or an artery only supplied by the extrahepatic collateral arteries) or unsuitable characteristics (neutrophil count < 1200/μL, platelet count < 60,000/μL, total bilirubin > 30 mmol/L, or albumin < 3.0 mg/dL). The study treatment was stopped if no recovery occurred after a 30-day delay.

If the study treatment was discontinued, the following treatment was defined as subsequent treatment. The subsequent treatment decisions of both groups would be made according to the same protocol by the same multidisciplinary team, based on the tumor burden, liver function, and the patient’s request. Basically, hepatic resections were performed on patients whose tumor shrank to be resectable. For patients with tumor progression without contraindications to TACE, repeating TACE was recommended. For patients whose residual tumors could not be embolized due to technical problems, radiofrequency ablations were used to destroy residual tumors when it was feasible. Conservative treatments were given to patients with terminal HCC, Child–Pugh C liver function, or Eastern Cooperative Oncology Group (ECOG) score > 2 [32].

### Efficacy and safety

According to the Response Evaluation Criteria in Solid Tumors (RECIST) [33], tumor responses were evaluated by radiologists who were blinded to the treatment. All objective responses were confirmed at least 4 weeks after the first observation. DCR is defined as the rate of complete response (CR) plus partial response (PR) plus stable disease (SD). Objective response rate (ORR) is defined as the rate of CR plus PR. TTP was calculated from the time of the first transcatheter therapy to the time of disease progression.

AEs and serious adverse events (SAEs) were monitored and recorded by professional nurses who were blinded to the treatment. Until study treatment was discontinued, AEs were assessed during and after each treatment procedure and at all follow-up visits and were graded according to the National Cancer Institute Common Terminology Criteria for Adverse Events (NCI-CTCAE) version 3.0 [34]. SAEs included treatment-related hospitalization or prolonged hospitalization, dysfunction or disability, life-threatening consequences, and death. Treatment-related death was defined as death caused by a complication within 30 days after the procedure.

### Follow-up

The follow-up ended on January 24, 2017. Contrast-enhanced computed tomography (CT) was performed every 6 weeks. Blood tests, including liver function test and the serum alpha-fetoprotein (AFP) level test, were performed during each treatment period. For patients with tumors that shrank to a resectable size, the choice of the next treatment was determined according to the patient’s request and the results of discussions by our multidisciplinary team.

### Sample size

The sample size was computed using the DCR as the main end point. A 5% significance level for the two-sided Fisher’s exact test for equality should have 80% power to detect differences between an 80% DCR with HAIC and a 50% DCR with TACE. The minimal sample size needed to detect a significant difference was calculated to be 38 patients per group.

### Statistical analyses

For comparisons of baseline variables, Student’s t test was used for continuous variables, and the Chi square test was used for categorical variables. TTP was estimated using the Kaplan–Meier method and compared using the log-rank test. The patients without tumor progression were censored. A *P* value < 0.05 was considered significant. All statistical processing was performed using the Statistical Package for Social Sciences version 13.0 (SPSS Inc., Chicago, IL, USA).

## Results

### Patient characteristics

**Fig. 1:**
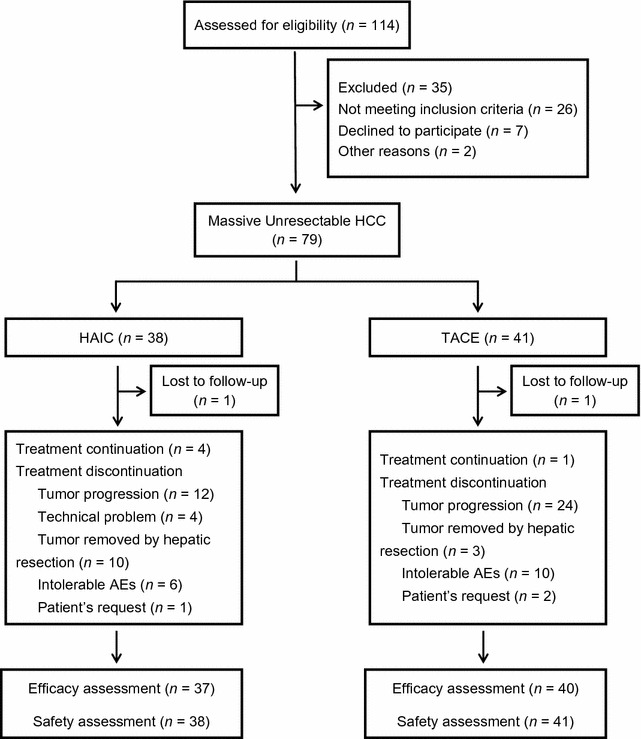
Flow diagram of patients with unresectable hepatocellular carcinoma who underwent either HAIC or TACE. *HAIC* hepatic artery infusion chemotherapy; *TACE* transarterial chemoembolization; *AEs* adverse events

Between October 1, 2015 and October 1, 2016, 114 consecutive patients with HCC were treated using either HAIC or TACE. Seventy-nine patients met the criteria for inclusion in this analysis: 38 underwent HAIC, and 41 underwent TACE (Fig. 1). The baseline characteristics of all patients are described in Table 1. The only difference between groups was that the HAIC group had more patients with a prolonged prothrombin time (*P* = 0.003).

**Table 1:** Baseline characteristics of patients with unresectable hepatocellular carcinoma who underwent either HAIC or TACE

Variable	HAIC [cases (%)]	TACE [cases (%)]	*P*
Total	38	41	
Age (years)			0.638
≤ 60	27 (71.1)	27 (65.9)	
> 60	11 (28.9)	14 (34.1)	
Gender			0.215
Male	30 (78.9)	37 (90.2)	
Female	8 (21.1)	4 (9.8)	
Tumor size (cm)			1.000
< 10	12 (31.6)	12 (29.3)	
≥ 10	26 (68.4)	29 (70.7)	
Tumor number			0.654
≤ 3	18 (47.4)	17 (41.5)	
> 3	20 (52.6)	24 (58.5)	
BCLC stage			0.338
A	15 (39.5)	11 (26.8)	
B	23 (60.5)	30 (73.2)	
Liver cirrhosis			0.368
No	20 (52.6)	26 (63.4)	
Yes	18 (47.4)	15 (36.6)	
Neutrophil: lymphocyte ratio			0.361
≤ 3	22 (57.9)	28 (68.3)	
> 3	16 (42.1)	13 (31.7)	
Hemoglobin (g/L)			1.000
< 100	1 (2.6)	1 (2.4)	
≥ 100	37 (97.4)	40 (97.6)	
Platelet count			1.000
< 100 × 10^9^/L	1 (2.6)	2 (4.9)	
≥ 100 × 10^9^/L	37 (97.4)	39 (95.1)	
Hepatitis B surface antigen			0.434
Negative	2 (5.3)	5 (12.2)	
Positive	36 (94.7)	36 (87.8)	
HBV DNA (IU/mL)			0.593
≤ 1000	10 (26.3)	8 (19.5)	
> 1000	28 (73.7)	33 (80.5)	
PT (s)			0.003
≤ 13.5	28 (73.7)	40 (97.6)	
> 13.5	10 (26.3)	1 (2.4)	
ALT (U/L)			1.000
≤ 40	17 (44.7)	18 (43.9)	
> 40	21 (55.3)	23 (56.1)	
ALB (g/L)			0.655
≤ 40	20 (52.6)	19 (46.3)	
> 40	18 (47.4)	22 (53.7)	
TBIL (μmol/L)			0.284
≤ 20.5	27 (71.1)	34 (82.9)	
> 20.5	11 (28.9)	7 (17.1)	
AFP (ng/mL)			0.813
≤ 400	12 (31.6)	15 (36.6)	
> 400	26 (68.4)	26 (63.4)	

*HAIC* hepatic artery infusion chemotherapy; *TACE* transarterial chemoembolization; *BCLC* Barcelona Clinic Liver Cancer; *HBV* hepatitis B virus; *PT* prothrombin time; *ALT* alanine transaminase; *ALB* albumin; *TBIL* total bilirubin; *AFP* alpha-fetoprotein

*P* values were calculated using a two-sided Chi square test

### Treatment

The 79 patients underwent a total of 215 study treatment sessions. Patients in the HAIC group received more sessions than did patients in the TACE group (146 vs. 69, *P* = 0.003). Patients in the HAIC group received an average of 3.8 ± 1.5 HAIC sessions (range 1–6 sessions; median: 4 sessions), whereas patients in the TACE group received an average of 1.7 ± 0.8 TACE sessions (range 1–3 sessions; median: 1 session) (*P* < 0.001). More patients in the HAIC group than in the TACE group underwent surgical resection (10 vs. 3, *P* = 0.033). There was no significant difference in the number of patients receiving other subsequent treatments between the HAIC and TACE groups (Table 2).

**Table 2:** Number of patients who received subsequent treatments in the HAIC or TACE group

Subsequent treatment	HAIC group	TACE group	*P*
Resection	10	3	0.033
Ablation	1	2	1.000
HAIC	3	1	0.347
TACE	4	3	0.705

*HAIC* hepatic artery infusion chemotherapy; *TACE* transarterial chemoembolization

### Safety

AEs are summarized in Table 3. There was no significant difference in the overall number of patients who had AEs between the HAIC and TACE groups (35 vs. 41, *P* = 0.107). However, the number of patients reporting AEs of grade ≥ 3 or SAEs was significantly smaller in the HAIC group than in the TACE group (AEs of grade ≥ 3: 13 vs. 27, *P* = 0.007; SAEs: 6 vs. 15, *P* = 0.044). Two patients in the TACE group died of an SAE, whereas no treatment-related death occurred in the HAIC group (*P* = 0.494). The occurrence rates of fever, hyperbilirubinemia, and alanine transaminase (ALT) elevation were higher in the TACE group, whereas those of sensory neuropathy, diarrhea, and hypoproteinemia were higher in the HAIC group (all *P* < 0.05). Furthermore, particular abdominal pain was observed in 10 patients of HAIC group when injecting OXA, and the pain disappeared when the injection was stopped.

**Table 3:** Study treatment-related AEs for patients with unresectable hepatocellular carcinoma who underwent either HAIC or TACE

Adverse event	Any grade (cases)			Grade 3–4 (cases)		
	HAIC group	TACE group	*P*	HAIC group	TACE group	*P*
Total	35	41	0.110	13	27	0.007
Postembolization syndrome						
Fever	7	30	< 0.001	0	8	0.006
Pain	30	27	0.219	1	1	1.000
Vomiting	21	18	0.371	4	1	0.190
Liver dysfunction						
Elevated ALT level	27	38	0.017	1	16	< 0.001
Hypoalbuminemia	34	27	0.016	0	0	1.000
Hyperbilirubinemia	13	35	< 0.001	0	3	0.241
Systemic toxicity						
Leukopenia	13	8	0.203	3	1	0.347
Neutropenia	6	6	1.000	0	1	1.000
Anemia	27	25	0.477	1	2	1.000
Thrombocytopenia	13	17	0.643	2	4	0.676
Anorexia	30	29	0.447	0	0	1.000
Diarrhea	12	1	0.001	1	0	0.481
Sensory neuropathy	9	1	0.006	1	0	0.481
Ascites/pleural effusion	2	3	1.000	1	2	1.000
Cholangitis	0	3	0.241	0	2	0.494
Hepatapostema	0	1	1.000	0	1	1.000
Hepatic failure	0	1	1.000	0	1	1.000
Renal failure	2	5	0.434	0	2	0.494
Gastrointestinal bleeding	1	2	1.000	1	2	1.000

*AE* adverse event; *HAIC* hepatic artery infusion chemotherapy; *TACE* transarterial chemoembolization; *ALT* alanine transaminase

*P* values were calculated using a two-sided Chi square test

The tolerability of HAIC and TACE was analyzed. In the TACE group, 10 (24.4%) patients had treatment terminated early (6 weeks after beginning therapy) due to intolerable chemoembolization-related AEs or withdrawal of consent by the patient. However, in the HAIC group, only 2 (5.3%) patients had treatment terminated early. This difference was significant (*P* = 0.026).

### Efficacy

The tumor responses among patients are shown in Table 4. No complete responses were achieved in either group. The DCR and ORR was significantly higher in the HAIC group than in the TACE group (DCR: 83.8% vs. 52.5%, *P* = 0.004; ORR: 54.1% vs. 9.8%, *P* < 0.001).

**Table 4:** Best tumor responses for patients with unresectable hepatocellular carcinoma who underwent either HAIC or TACE

Response	HAIC group [cases (%)]	TACE group [cases (%)]	*P*
Objective response	20 (54.1)	4 (9.8)	< 0.001
Disease control	31 (83.8)	21 (52.5)	0.004
Complete response	0	0	1.000
Partial response	20 (52.6)	4 (9.8)	< 0.001
Stable disease	11 (28.9)	17 (41.5)	0.347
Progressive disease	6 (15.8)	19 (46.3)	0.004
Not evaluable	1 (2.6)	1 (2.4)	

*HAIC* hepatic artery infusion chemotherapy; *TACE* transarterial chemoembolization

*P* values were calculated using a two-sided Chi square test

**Fig. 2:**
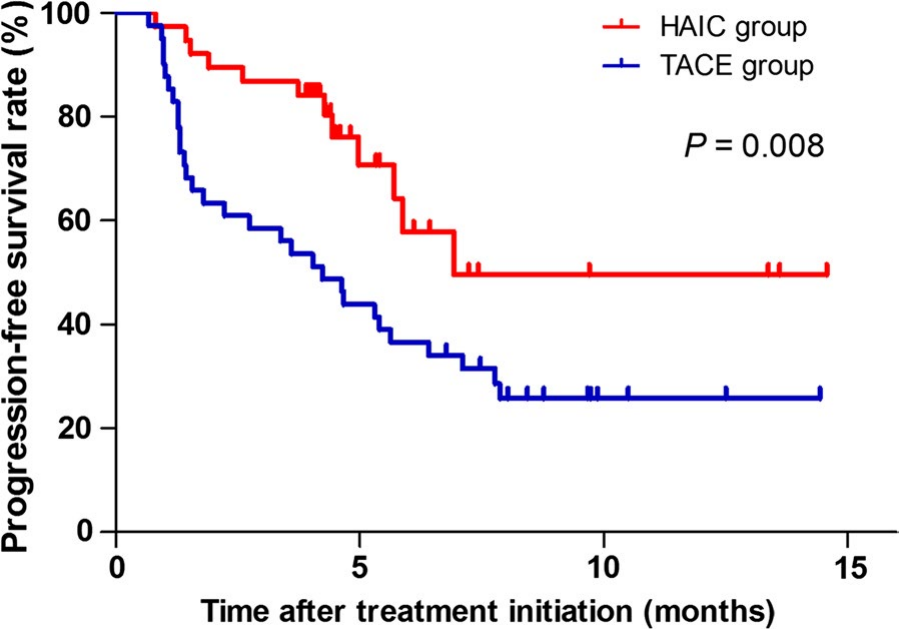
Kaplan-Meier estimated progression-free survival curves of patients with unresectable hepatocellular carcinoma. Of the 79 patients, 38 underwent HAIC, and 41 underwent TACE. *HAIC* hepatic artery infusion chemotherapy; *TACE* transarterial chemoembolization

In the univariate analysis, the median TTP was 5.9 months (95% confidence interval [CI] = 3.9–7.8 months) in the HAIC group and 3.6 months (95% CI = 1.8–5.4 months) in the TACE group. The TTP was significantly longer in the HAIC group than in the TACE group (*P* = 0.015), and the hazard radio was 2.35 (95% CI = 1.16–4.76). Progression-free survival was shown in Fig. 2.

## Discussion

In this prospective, non-randomized, phase II study, we compared the safety and efficacy of HAIC with mFOLFOX6 versus TACE in patients with massive unresectable HCC. This study demonstrated encouraging results for both safety and efficacy of HAIC with mFOLFOX6. HAIC-related AEs in our study were consistent with previous report investigating systemic chemotherapy with mFOLFOX6 [26] with the exception of one particular AE (abdominal pain) associated with OXA injection. The occurrence rate of cytotoxic agent-specific AEs was significantly higher in the HAIC group than in the TACE group, including sensory neuropathy (OXA-specific) and diarrhea (5-FU-specific). In contrast, the occurrence rate of embolization-specific AEs, such as fever and hyperbilirubinemia, was significantly higher in the TACE group than in the HAIC group. There were no significant differences in the overall rate of AEs between the two groups. However, the numbers of patients reporting AEs of grade ≥ 3 and SAEs were both significantly smaller in the HAIC group than in the TACE group. The tolerability of HAIC was also better than that of TACE. Compared with the HAIC group, more patients in the TACE group had their study treatment terminated early due to intolerable treatment-related AEs or the withdrawal of consent by the patient (10 vs. 2). These findings suggested a significant superiority in the safety of HAIC with mFOLFOX over TACE.

Some previous studies recommended using the modified RECIST (mRECIST) criteria to assess tumor response in clinical trials of locoregional treatment of HCC, because devascularization rather than tumor shrinkage may be a hallmark of response in HCC [35]. Nevertheless, in contrast to HAIC, the CT response assessment following TACE may be confounded by the presence of lipiodol. Therefore, RECIST is a better guideline than mRECIST for comparing tumor responses to HAIC and TACE, and we used RECIST to evaluate tumor responses in the present study. Patients in the HAIC group achieved a significantly higher partial response rate than did patients in the TACE group (52.6% vs. 9.8%). There was also a significant difference between the two groups in the median TTP (5.9 vs. 3.6 months). The number of patients who underwent resection was significantly larger in the HAIC group than in the TACE group (10 vs. 3). These findings suggested a significant superiority of HAIC with mFOLFOX over TACE in terms of efficacy on massive HCCs. Because more than half of the recruited patients remained alive when this study was terminated, an overall survival analysis could not be conducted. The above results strongly support the launch of a large prospective, randomized, controlled study as soon as possible.

The first limitation of the present study was its non-randomized nature because the treatment choices depended on the patients’ requests. However, no significant differences were found in the baseline characteristics between the two groups. Moreover, the patients in both groups were treated strictly according to the study protocol. The difference in the numbers of study treatment sessions can also be explained by the different treatment intervals and the superiority of HAIC with mFOLFOX over TACE in terms of both efficacy and safety. The second limitation was that subsequent treatments may be a confounding factor. However, subsequent treatments of both groups were performed according to the same multidisciplinary treatment protocol by the same team. Furthermore, we used tumor response to evaluate efficacy of study treatment, and we recorded study treatment-related AEs. Tumor responses and study treatment-related AEs were not likely be influenced by subsequent treatment.

In conclusion, the present study demonstrated that patients in the HAIC group had significantly better tumor responses and less SAEs than patients in the TACE group. More patients in the HAIC group than in the TACE group received potential curative treatments after tumor down-staging. Thus, HAIC might be a better choice for patients with massive unresectable HCC.
